# Understanding climate change knowledge and risk denial in a Southern Italian university population: a cross-sectional study

**DOI:** 10.3389/fpubh.2026.1733397

**Published:** 2026-01-30

**Authors:** Silvia Angelillo, Gianfranco Di Gennaro, Giuseppe Servello, Claudia Pileggi, Adele Sarcone, Carmelo G. A. Nobile

**Affiliations:** 1 Department of Health Sciences, School of Medicine, University “Magna Gracia” of Catanzaro, Catanzaro, Italy; 2 Department of Psychology and Health Sciences, Pegaso University, Naples, Italy; 3 CRUISE Research Center, Science of Health Department, University “Magna Gracia” of Catanzaro, Catanzaro, Italy; 4 Department of Pharmacy, Health and Nutritional Sciences, University of Calabria, Cosenza, Italy

**Keywords:** climate change, knowledge, one health, risk perception, university students

## Abstract

**Background:**

This study investigated the level of knowledge and risk perception related to climate change and its health impacts among a university population in Southern Italy.

**Methods:**

Data were collected through a paper-based questionnaire administered in classrooms and offices, covering sociodemographic characteristics, climate change knowledge, risk perception, environmental attitudes, and awareness of the “One Health” approach.

**Results:**

Among 551 participants, 57.2% achieved high knowledge, which was associated with older age, a climate-related academic or professional background, and attendance at the University of Catanzaro. Most respondents (96.3%) recognized the impact of global warming on human health, though 11% believed climate change severity was overstated, a view more common among men and married or separated individuals. Awareness of the “One Health” concept was limited to 41.4%, yet those familiar with it acknowledged its importance in preventing climate-related diseases. Internet and social media were the primary information sources.

**Conclusions:**

Findings reveal generally high awareness but notable variability across subgroups, highlighting the need for targeted educational interventions that combine scientific knowledge with environmental attitudes to promote effective mitigation and adaptation strategies.

## Introduction

Climate change refers to long-term alterations in temperature and weather patterns, which may occur naturally but are increasingly driven by human activity (1). Since the 19th century, the primary anthropogenic driver has been the combustion of fossil fuels, coal, oil, and gas, leading to greenhouse gas emissions that trap heat in the atmosphere and raise global temperatures (2).

The health impacts of climate change are broad and severe, encompassing cardiovascular, mental, infectious, and even oncological conditions (3–8). These effects extend beyond physical health, compromising safety, productivity, and wellbeing (9, 10). Vulnerable populations, particularly in low- and middle-income countries, are disproportionately affected due to weaker healthcare systems and limited adaptive capacity (11).

Climate change also contributes to human and animal migration, rising sea levels, extreme weather events, and the spread of vector-borne and waterborne diseases (12, 13). These global changes underscore the interconnectedness of environmental, human, and animal health, a concept formalized under the “One Health” approach. Promoted by the Food and Agriculture Organization (FAO), World Organization for Animal Health (WOAH), United Nations Environment Programme (UNEP), and World Health Organization (WHO), “One Health” recognizes that the health of people, animals, and ecosystems are deeply interdependent, and calls for interdisciplinary collaboration to address shared threats such as climate change, zoonoses, antimicrobial resistance, and pollution (14).

Despite growing scientific consensus and visible manifestations of climate change, public awareness and understanding of its health implications remain uneven. Europe has already experienced dramatic consequences. Indeed, the summer of 2022 over 20.000 excess deaths linked to heatwaves occurred. Not only, in 2023, Europe was among the world's top greenhouse gas emitters (15). Climate-related disasters, such as wildfires, droughts, and heatwaves, are becoming more frequent and intense, alongside the geographic expansion of diseases like dengue, West Nile virus, and leishmaniasis (12). Within the context of climate change, the One Health framework provides an essential interpretive lens, emphasizing that environmental degradation, shifts in ecosystems, and alterations in animal habitats directly and indirectly affect human health. The concept has expanded significantly over the past decade, stressing the need for improved public understanding, interdisciplinary approaches, and stronger integration into education and awareness initiatives (16). This assesses One Health awareness particularly relevant among younger generations and academic communities.

Understanding public knowledge and risk perception is essential to develop effective communication campaigns, inform policy, and promote adaptive and mitigative action (17). Without this foundation, efforts by governments, institutions, and communities to address the health effects of climate change risk falling short (18). Moreover, the economic burden of inaction is likely to be substantial, as recent studies estimate that for every 1 °C increase in global temperature, Gross Domestic Product (GDP) may decline by as much as 12% (19). Finally, it is important to keep in mind that global warming risk denial is widespread worldwide, and that informing the public about this topic can play a key role in addressing it (20).

Although numerous studies have examined climate change awareness in the general population, evidence focusing specifically on the university population in Europe remains limited (21–23). University populations are an important group, as they represent future professionals and decision-makers whose knowledge and attitudes will shape societal responses to the climate crisis. Even fewer investigations have jointly explored climate risk denial, sources of information, and awareness of the One Health framework within university populations, and the available evidence on these aspects remains fragmented across different study designs and settings.

This cross-sectional study aims to evaluate the level of knowledge about climate change and its health impacts among a university population in Southern Italy, comprising both students and university staff. A further key objective is to investigate the prevalence of climate change denial or underestimation of its associated health risks. In addition, the study explores attitudes toward mitigation and adaptation strategies, awareness of the “One Health” approach, and the sources of information most frequently consulted by participants.

## Methods

Data were collected through paper-based questionnaires administered in university classrooms and academic offices using a convenience sampling approach. Classes and offices were selected based on accessibility and availability at the time of data collection, following agreement with teaching staff. The survey instrument consisted of three main sections and was administered in paper format, completed in person by participants in university classrooms and offices. All individuals present at the time of data collection who met the inclusion criteria were invited to participate. Because questionnaires were distributed during in-person sessions and the total number of eligible individuals present at the time of administration could not be formally recorded, a precise response rate could not be calculated. Participation was entirely voluntary, and each copy included an informed consent form, which was signed by all participants prior to their involvement in the study.

Section I gathered sociodemographic data, including age, sex, marital status (categorized as single, married/cohabiting, or separated/divorced/widowed), occupational status (unemployed, student, employed, or self-employed), and educational attainment, defined as the highest completed qualification at the time of the survey (high school diploma or university degree).

Section II focused on climate change knowledge and perception. It included 15 multiple-choice questions assessing knowledge of causes and health consequences of climate change, with an option for “I don't know,” which was scored as incorrect. This section also contained items exploring risk perception, awareness of the “One Health” approach, identification of vulnerable populations, contributing factors to climate change, and main sources of information. Two questions were specifically designed to detect elements of climate change denial or risk underestimation.

Section III addressed environmental attitudes through eight items assessing agreement with statements about individual and collective actions to mitigate climate change. This section concluded with two items gauging participants' interest in further education on the topic.

Prior to data collection, the questionnaire was pretested to assess clarity and content relevance, leading to minor refinements. The instrument was developed specifically for this study, drawing on items used in previous surveys on climate change knowledge, risk perception, and environmental attitudes. It was intended for exploratory and descriptive purposes rather than as a psychometric scale; therefore, formal reliability analyses (e.g., Cronbach's alpha) were not performed.

### Statistical analysis

Data were analyzed using Stata version 17 (www.stata.com). Continuous variables were summarized as means and standard deviations when normally distributed, and as medians and interquartile ranges (IQR) in case of skewed distributions. Categorical variables were presented as counts and percentages.

### Primary analysis

The primary outcome was the knowledge score regarding climate change and global warming, calculated as the total number of correct responses to the 15 items in Section 2.3 of the questionnaire. Responses coded as “Don't know” were considered incorrect. The resulting score ranged from 0 to 15, with higher scores indicating greater knowledge. To facilitate interpretation and in line with previous studies, the score was dichotomized as high or low using a 70% cut-off (≥11/15 correct answers) (17, 24).

The association between knowledge level (high vs. low) and categorical variables was assessed using the chi-squared test, while the Wilcoxon–Mann–Whitney test was used to compare age distributions between the two groups.

A logistic regression model was then used to identify independent predictors of high knowledge. All sociodemographic variables considered in the bivariate analysis were included in the multivariable logistic regression model, regardless of their statistical significance, in order to adjust for potential confounding factors and to reflect their theoretical relevance.

### Secondary analyses

As secondary outcomes, two key items were considered as potential indicators of climate change denial or risk underestimation: item 2.2 (“Do you think global warming has an impact on human health?”) and item 2.8 (“Do you think climate change is actually less serious than scientists claim?”). Their association with the knowledge score and with sociodemographic variables was assessed using the aforementioned logistic regression analysis.

Additional analyses focused on responses to other sections of the questionnaire. Perceptions about climate change (Section 2.1–2.2), awareness of the “One Health” approach (Section 2.4), and responses to multiple-response items on vulnerable populations, contributing factors, and sources of information (Items 2.5–2.7) were analyzed descriptively. For each item, the number and percentage of participants selecting each option were reported.

All statistical tests were two-tailed, and statistical significance was set at *p* < 0.05.

## Results

### Sample characteristics

The final sample included 551 participants with a median age of 20 years (IQR: 19–24). Most respondents were female (80.5%), single (87.9%), and students (93.4%). A high school diploma was the most common educational qualification (90.9%). A total of 65.5% of participants reported either studying or working in a field related to climate change. More than half (56%) were enrolled at the University of Cosenza, while the remaining 44% attended the University of Catanzaro.

### Knowledge score and associated factors

Participants' knowledge scores, based on the number of correct responses to 15 items, ranged from 0 to 15. The distribution was skewed, with a median of 12 (IQR: 10, 13). Based on a 70% cut-off (≥11 correct answers), 57.2% of respondents were classified as having high knowledge.

In bivariate analyses, high knowledge was significantly associated with older age (median 21 vs. 20 years; *p* = 0.015), being a student (57.8 vs. 37.8% among non-students; *p* = 0.018), studying or working in a climate-related field (64.2 vs. 41.8%; *p* < 0.001), and attending the University of Catanzaro rather than the University of Cosenza (67.3 vs. 47.9%; *p* < 0.001).

Although the proportion of high knowledge appeared greater among men (63.6%) compared to women (54.7%), the difference did not reach statistical significance (*p* = 0.092). Likewise, no statistically significant associations were observed with marital status (*p* = 0.161) or educational level (*p* = 0.214), although participants with a university degree showed a slightly higher proportion of high knowledge (64.7%) than those with a high school diploma (55.7%).

In the multivariable logistic regression model, high knowledge remained significantly associated with having a study or work background related to climate change (OR = 1.77; 95% CI: 1.14, 2.75; *p* = 0.011) and with being enrolled at the University of Cosenza (OR = 0.59; 95% CI: 0.38, 0.92; *p* = 0.020), indicating lower odds of high knowledge compared to participants from the University of Catanzaro. Participants who were married or cohabiting also had significantly higher odds of high knowledge compared to single individuals (OR = 2.06; 95% CI: 1.08, 3.92; *p* = 0.028).

No significant associations were observed for sex, age, work status, or highest level of education attained. The model showed good overall fit (*p* = 0.213). Multicollinearity among covariates included in the multivariable logistic regression models was assessed and no evidence of problematic collinearity was observed. Model assumptions for logistic regression were evaluated and were considered to be reasonably met.

### Climate change denial and risk perception

#### Perceptions of climate change and health impacts

Overall, the vast majority of participants (96.3%) agreed that global warming has an impact on human health. In bivariate analyses, significant differences emerged across some sociodemographic variables. Female participants were more likely than males to recognize this health impact (97.1 vs. 92.7%, *p* = 0.029). Marital status also showed a significant association (*p* = 0.002), with the lowest agreement observed among separated/divorced/widowed individuals (71.4%) compared to singles (96.6%) and those married or cohabiting (96.7%). No statistically significant differences were found with respect to age (*p* = 0.306), work status (*p* = 0.146), educational attainment (*p* = 0.484), university attended (*p* = 0.737), climate-related work/study background (*p* = 0.196), or knowledge score (*p* = 0.404).

In the multivariable logistic regression model, increasing age was independently associated with a higher likelihood of perceiving global warming as a threat to human health (OR = 1.18; 95% CI: 1.02, 1.37; *p* = 0.029). Female sex also showed higher odds compared to males (OR = 2.52; 95% CI: 0.87, 7.27), although this association did not reach statistical significance (*p* = 0.087). Participants who were separated, divorced, or widowed were less likely to recognize the health impact of climate change (OR = 0.06; 95% CI: 0.007, 0.56; *p* = 0.130), while those in the “married/cohabiting” category showed no significant difference (OR = 0.64; 95% CI: 0.12, 3.36; *p* = 0.602).

A marginal association was observed for work status, with non-students showing lower odds of perceiving climate change as a health threat compared to students (OR = 0.21; 95% CI: 0.04, 1.02; *p* = 0.053). No significant associations were found for education level (OR = 0.81; 95% CI: 0.08, 7.89; *p* = 0.858), university attended (OR = 3.00; 95% CI: 0.78, 11.59; *p* = 0.111), or work/study background in a climate-related field (OR = 2.49; 95% CI: 0.66, 9.42; *p* = 0.180). Additionally, the knowledge score was not independently associated with the outcome (OR = 1.22; 95% CI: 0.48, 3.12; *p* = 0.672).

#### Perceptions of climate change severity

In total, 62 participants (11.0%) agreed with the statement that climate change is actually less serious than scientists claim. This belief was more common among men than women (20.0 vs. 8.8%, *p* = 0.001), and among participants who were married, cohabiting (21.3%), or separated/divorced/widowed (42.9%) compared to those who were single (9.3%, *p* < 0.001). No significant differences in this perception were observed based on age (*p* = 0.793), work status (*p* = 0.615), education level (*p* = 0.112), university attended (*p* = 0.369), or whether participants studied or worked in climate-related fields (*p* = 0.341). Similarly, knowledge level about climate change was not significantly associated with the likelihood of endorsing this skeptical view (*p* = 0.790).

In the multivariable logistic regression model, several sociodemographic characteristics were independently associated with the belief that climate change is less serious than scientists claim. Women had significantly lower odds of endorsing this belief compared to men (OR = 0.34; 95% CI: 0.18, 0.66; *p* = 0.001). Participants who were married or cohabiting (OR = 2.76; 95% CI: 1.24, 6.12; *p* = 0.012) and those who were separated, divorced, or widowed (OR = 10.81; 95% CI: 1.91, 61.05; *p* = 0.007) showed higher odds of skepticism compared to single individuals. No significant associations were found for age (OR = 0.98; 95% CI: 0.92, 1.04; *p* = 0.448), university attended, employment status, education level, or climate-related study/work background. Likewise, knowledge score was not significantly associated with this perception (OR = 1.03; 95% CI: 0.58, 1.81; *p* = 0.932).

### Perceptions of climate change and awareness of the one health approach

All participants (100%) reported having heard about climate change and/or global warming.

Regarding the belief that global warming has an impact on human health (CCsalute), the vast majority of respondents answered affirmatively (96.3%), while 0.9% responded negatively and 2.8% were uncertain.

Among the 551 participants, 41.4% reported being familiar with the concept of “One Health.” Among those aware of the concept (*n* = 233), nearly all (98.7%) agreed that climate change poses a threat not only to human health but also to animal and environmental health. Furthermore, 85.0% believed that the “One Health” approach could help prevent climate change-related diseases, while 13.7% were uncertain and only 1.3% disagreed.

### Perceptions of populations most vulnerable to climate change

When asked “In your opinion, who is most vulnerable to the effects of global warming and climate change?”, the majority of participants (84.6%) selected “The entire population.” Other frequently identified vulnerable groups included “People living in poverty or facing social disadvantage” (30.7%), “Residents on the coast or in flood-prone areas” (27.2%), “People with pre-existing illnesses” (26.3%), and “Children and adolescents” (25.0%). Smaller proportions of respondents indicated “Outdoor workers” (22.9%) and “People with fair or sensitive skin” (15.5%) as being particularly susceptible to climate-related health risks.

### Perceptions of factors contributing to climate change

When asked which factors may contribute to global warming and climate change, the vast majority of respondents (89.9%) selected “Carbon emissions from vehicles and industrial emissions.” A smaller proportion identified “Ozone depletion” (61.5%) and “Deforestation” (58.3%) as contributing factors. Only 3.7% attributed climate change to “God's will,” and 22.2% selected “Other factors.”

### Sources of information on climate change

When asked “What are your sources of information about global warming and climate change?”, the vast majority of participants (88.8%) reported “Internet and social media (Facebook, Twitter, blogs…”) as their primary source. Other frequently cited sources included “Mass media (TV, radio, newspapers)” (71.4%) and, to a lesser extent, “Websites of the government and official institutions” (33.0%). Less commonly reported sources were “Books, educational programs, and conferences” (24.3%), “I am studying it at school or university” (24.9%), and “Family and friends” (16.5%).

## Discussion

This study examined the level of knowledge and attitudes toward climate change among a large sample of university students and workers. The findings indicate that, while general awareness is high, substantial variability remains in factual knowledge and in the perception of climate-related risks across subgroups. These findings of the current survey are in line with other experiences among university students which reported a strong concern about climate change coexists with heterogeneous levels of factual knowledge. Similar results have been reported in France and Greece, where moderate awareness and discipline-related differences in engagement have been observed (25). Evidence from Germany also suggests that higher knowledge does not necessarily correspond to higher climate risk perception, as affective and cultural factors play a significant role in shaping climate risk perception (26).

Using a 70% cut-off to define a high level of knowledge, just over half of the respondents met this threshold. Factors significantly associated with high knowledge included older age and a background in health or life sciences. There is no rigorous agreement in the literature on the importance of advanced age as a predictor of knowledge. For example, a study conducted in El-Beheira (Egypt) suggested that advanced age is associated with greater knowledge. However, an international survey showed that young citizens are more aware of and concerned about climate change issues (27, 28). Similarly, it is difficult to conclude that health background is a possible and certain predictor of knowledge. In some studies, this association is confounded, while in other cases, the difference in knowledge between students with different backgrounds does not reach statistical significance (29–31).

Interestingly, participants enrolled at the University of Cosenza were significantly less likely to reach the high knowledge threshold than those at the University of Catanzaro. While the underlying reasons are unclear, this difference may reflect variations in curricula, exposure to public health or planetary health topics, or local academic initiatives.

Despite nearly universal agreement that global warming poses a threat to human health, around one in 10 respondents expressed the belief that the severity of climate change is overstated. This view, often considered a marker of climate change denial, was more common among males, older participants, and those who were married or divorced. These observations are supported by studies highlighting a greater tendency to underestimate the severity of climate change among men and older age groups, consistent with prior research linking denialism to sociodemographic factors such as gender and age, as well as political orientation and perceived personal vulnerability (32–34). However, it is important to note that some research presents less consistent findings regarding the influence of these sociodemographic characteristics, suggesting the need for further investigation to fully understand the role of this variable in climate change beliefs (35). Awareness of the “One Health” framework was limited: fewer than half of the respondents had heard of it. This result indicates that participants may not yet fully integrate the ecological dimension of health into their understanding of climate-related risks. This gap is particularly relevant considering the increasing burden of vector-borne diseases, ecosystem disruptions, and climate-driven interactions between human and animal health. However, among those familiar with the concept, there was strong agreement on its relevance to global health and disease prevention. The low percentage of individuals aware of the concept of “One Health” is consistent with findings from a Chinese study, where approximately 40% of respondents reported being aware of it (36). Comparable results have been reported in recent Italian research, which found that university students frequently underestimate or only partially understand the health consequences of climate change (22). When considered together with the Chinese data, this suggests that the limited familiarity with the One Health framework observed in our sample is likely part of a broader international gap in ecological health literacy rather than a context-specific issue.

The study also found a robust association between environmental attitudes and climate change knowledge. Participants with more favorable environmental views were significantly more likely to achieve high knowledge scores. This suggests that educational interventions addressing both cognitive and affective components may be more effective in promoting climate action.

### Strengths and limitations

This study benefits from a large sample size and a comprehensive questionnaire that addresses both objective knowledge and subjective attitudes. However, several limitations must be acknowledged. First, the cross-sectional design precludes any causal inference. Second, the sample was geographically limited to Southern Italy and included students from only two universities in the same region. As climate change attitudes and knowledge may vary across different geographical, socio-cultural, and institutional contexts, caution is required when generalizing these findings to the broader Italian university population. In addition, the use of a convenience sampling approach and the absence of a formally calculable response rate limit the assessment of sample representativeness. Third, self-reported attitudes are potentially subject to social desirability bias. Fourth, the dichotomization of the knowledge score, although consistent with previous studies and useful for interpretation, remains an arbitrary choice. Finally, the sample showed an unbalanced gender distribution, with a predominance of female respondents. This reflects typical participation patterns in online surveys and in certain academic fields, but it may limit the generalizability of the results, particularly regarding gender-related differences in climate change perceptions. Moreover, the limited number of participants in some sociodemographic subgroups reduced the precision of certain regression estimates, as indicated by wide confidence intervals. Finally, the questionnaire was developed for exploratory purposes and did not undergo formal psychometric validation, which should be considered when interpreting the findings. Considering these limitations, the findings should be considered exploratory and hypothesis-generating, warranting confirmation through validated instruments and specifically designed studies.

## Conclusions

Promoting climate change literacy among university students, especially those outside scientific disciplines, should be a public health and educational priority. Interdisciplinary strategies that incorporate the health dimensions of climate change and frameworks such as One Health could enhance both knowledge and engagement. The limited awareness of the One Health framework further indicates a gap in understanding the interconnected nature of environmental, animal, and human health. This reinforces the need for integrated educational approaches capable of addressing the complex drivers of climate-related health risks.

## Data Availability

The raw data supporting the conclusions of this article will be made available by the authors, without undue reservation.
